# Identification of Potential HCV Inhibitors Based on the Interaction of Epigallocatechin-3-Gallate with Viral Envelope Proteins

**DOI:** 10.3390/molecules26051257

**Published:** 2021-02-26

**Authors:** Fareena Shahid, Noreen, Roshan Ali, Syed Lal Badshah, Syed Babar Jamal, Riaz Ullah, Ahmed Bari, Hafiz Majid Mahmood, Muhammad Sohaib, Siddique Akber Ansari

**Affiliations:** 1 Institute of Basic Medical Sciences, Khyber Medical University, Peshawar 25000, Pakistan; fareenashahid1@gmail.com (F.S.); noreenkhattak777@gmail.com (N.); Roshanali.ibms@kmu.edu.pk (R.A.); 2 Department of Chemistry, Islamia College University, Peshawar 25120, Pakistan; shahbiochemist@gmail.com; 3 Department of Biological Sciences, National University of Medical Sceinces, Rawalpindi 46000, Pakistan; babar.jamal@numspak.edu.pk; 4 Department of Pharmacognosy (MAPPRC), College of Pharmacy, King Saud University, Riyadh 11451, Saudi Arabia; 5 Department of Pharmaceutical Chemistry, College of Pharmacy, King Saud University, Riyadh 11451, Saudi Arabia; abari@ksu.edu.sa (A.B.); sansari@ksu.edu.sa (S.A.A.); 6 Department of Pharmacology, College of Pharmacy, King Saud University, Riyadh 11451, Saudi Arabia; harshad@ksu.edu.sa; 7 Department of Soil Science, College of Food and Agriculture Sciences, King Saud University, P.O. Box 2460, Riyadh 11451, Saudi Arabia; msohaib@ksu.edu.sa

**Keywords:** hepatitis C virus, E2 protein, homology modeling, epigallocatechin-3-gallate, virtual screening

## Abstract

Hepatitis C is affecting millions of people around the globe annually, which leads to death in very high numbers. After many years of research, hepatitis C virus (HCV) remains a serious threat to the human population and needs proper management. The in silico approach in the drug discovery process is an efficient method in identifying inhibitors for various diseases. In our study, the interaction between Epigallocatechin-3-gallate, a component of green tea, and envelope glycoprotein E2 of HCV is evaluated. Epigallocatechin-3-gallate is the most promising polyphenol approved through cell culture analysis that can inhibit the entry of HCV. Therefore, various in silico techniques have been employed to find out other potential inhibitors that can behave as EGCG. Thus, the homology modelling of E2 protein was performed. The potential lead molecules were predicted using ligand-based as well as structure-based virtual screening methods. The compounds obtained were then screened through PyRx. The drugs obtained were ranked based on their binding affinities. Furthermore, the docking of the topmost drugs was performed by AutoDock Vina, while its 2D interactions were plotted in LigPlot+. The lead compound mms02387687 (2-[[5-[(4-ethylphenoxy) methyl]-4-prop-2-enyl-1,2,4-triazol-3-yl] sulfanyl]-N-[3(trifluoromethyl) phenyl] acetamide) was ranked on top, and we believe it can serve as a drug against HCV in the future, owing to experimental validation.

## 1. Introduction

E2 protein is usually considered as the site for HCV entrance because it contains highly conserved regions [1]. Generally, it has a role in target cell recognition and its attachment with the virus. The major variations in E2 protein are highly observed in hypervariable regions. Three different hypervariable regions have been reported recently. Hypervariable region 1 has a role in target cell recognition and its attachment. Hypervariable region 2 usually helps in binding with the receptors of the cell surface [2,3]. Therefore, the diverse nature of the virus and certain drawbacks present in the available treatment compelled scientists to identify a drug that is cost-effective and pan-genotypic in nature.

Epigallocatechin-3-gallate is a component of green tea. It contains some other catechins too, such as epigallocatechin-gallate 46.8%, epicatechin gallate 13.54%, epigallocatechin 2.28%, epicatechin 8.07%, and gallocatechin 7.24%. Certain flavanols are also present in small amounts [4]. It is capable of inhibiting the HCV as approved by means of cell culture analysis [5,6,7]. They also showed that it can specifically target the virus entry into the cell, as well as its attachment and transmission from one cell to another. Therefore, the procedure of de novo drug synthesis was used to evaluate its effects on HCV envelope proteins.

The application of computational techniques in the field of biological sciences helped provide new approaches in drug development and designing. Computer-aided drug designing can assist in accelerating the process of therapeutic drug synthesis, which requires a wet lab and screening process that are costly and time-consuming. The advent of revolutionary drug development, such as virtual screening, homology modeling, genomics, proteomics, and de novo synthesis drastically increased the process of drug development [8,9,10]. The two databases named ZINC and PubChem contain millions of purchasable “drug-like” compounds, effectively all organic molecules that are for sale, a quarter of which are available for immediate delivery. They connect purchasable compounds to high-value ones, such as metabolites, drugs, natural products, and annotated compounds from literature. They also offer new analysis tools that are easy for non-specialists yet with few limitations for experts. These databases retain their original 3D roots, and all molecules are available in biologically relevant, ready-to-dock formats. Thus, these databases are useful sources of ligand screening [11,12]. Calculation of logP, polar surface area (PSA), molecular weight, number of hydrogen-bond donors and acceptors, and number of rotatable bonds are the criteria for selection of drug-like molecules obtained from these databases [13]. The molecules in these databases are applied in virtual screening for identification of their inhibitory action against target structures [14].

Globally, 170 million people are infected with hepatitis C virus. Approximately 15–20% of the population progress to chronic liver infection in 15 to 20 years [15]. Hepatitis C virus is an RNA virus that belongs to the family of Flaviviridae having a genus of hepacivirus. The enveloped genome of HCV is positive-sense having 3010 amino acids and 9600 nucleotide bases. The HCV structure contains Open Reading Frame, 5′ non-coding region and 3′ untranslated region. ORF region encodes 11 proteins commonly known as structural and non-structural proteins. Structural proteins are E1, E2, and p7, while non-structural proteins are NS2, NS3, NS4A, NS4B, NS5A, and NS5B [16].

The aim of the present study is to identify potential HCV inhibitors based on their interaction with Epigallocatechin-3-gallate by using ligand-based virtual screening as well as target-based virtual screening. Virtual screening helps in evaluating various scaffolds of the molecule such as its interaction energy and binding energies, etc. Conventional drug development can cause toxicity in the host, while the in-silico approach abrogates the toxic effect on host cells.

## 2. Materials and Methods 

### 2.1. Homology Modeling

The E2 model of HCV was designed by homology modelling. A representative sequence for each genotype was retrieved from the UniProt database [17]. The homology modelling of these retrieved sequences produced 164 models that were built via online homology modelling servers, i.e., SWISS-MODEL [18], I-TASSER [19], LOMETS [20], CPH models [21], as well as MODELLER [22]. The template used for this purpose was 4MWF. The models obtained were further evaluated on ProCheck [23] and ProSa [24] to analyze the stereochemical properties of protein structures. The selected models were energy-minimized and refined with ModRefiner [25] to ensure that the confirmations obtained were stable in nature. The finest possible model obtained was selected for further analysis. The examination of the models was done on Discovery Studio Visualizer [26].

### 2.2. Binding Site Prediction

The binding sites of the model were predicted through different sites such as COACH [27], TM-SITE, S-SITE [28], CO-FACTOR [29], FIND-SITE, and CON-CAVITY [30]. The pockets having the highest C-Score were then further compared with binding sites predicted in literature. The pockets that were predicted by both the literature and online tools were further selected for virtual screening.

### 2.3. Ligand-Based Virtual Screening 

The screening of ligands was done on three commercially available online servers such as ZINC [11], PubChem [12], and DrugBank [31]. The screening of the structurally-similar ligands with the known inhibitor, EGCG, was done by following 70% similarity index for ZINC, 3D similarity search for Pubchem, and 50% cutoff value for Drug Bank. The ligands identified twice were considered only once. The drug-like properties of the ligands were evaluated using Lipinski’s Rule of Five, while the toxicity filters were employed with the help of server Swiss ADME [32].

### 2.4. Structure-Based Virtual Screening

Dockblaster [33], pep mms mimic [34], and MTiOpenScreen [35] were used for drug mining against the E2 protein of HCV to find its potential inhibitors. The residues bind within the specific binding cavities. The drug’s likeliness of the ligand was evaluated while the toxicity filters were applied. The ligands fulfilling all the properties were further selected for docking.

### 2.5. Library Designing

The library of the lead molecule was designed with CLEVER [36], which helps in analyzing chemical compounds as well as the conversion of the lead molecule’s chemical format.

### 2.6. Virtual Screening and Docking on PyRX

PyRx [37] is a graphical interface for users to execute virtual screening. It can evaluate the binding affinity as well as the RMSD scores of each ligand. The library of the ligands was subjected to virtual screening against the E2 protein of HCV. Docking of the selected ligands was carried out on an automated docking tool AutoDock Vina [38]. It performed ligand docking with protein in a specific grid. The tool helped in protein and ligand preparation, optimization, and grid generation near the active sites and then docking. 

### 2.7. Docking

The interaction of the obtained ligands with the protein was analyzed with AutoDock Vina using PyRX. The docking was done on the specific pockets that were predicted by literature and databases.

### 2.8. Analysis of Interaction

To analyze the interaction between docked ligands and protein, LigPlot+ [39] was used. This helped us to clearly observe the type of bonding between the ligands and protein.

## 3. Results and Discussion

### 3.1. Sequences Obtained after Alignment

**Figure 1:**
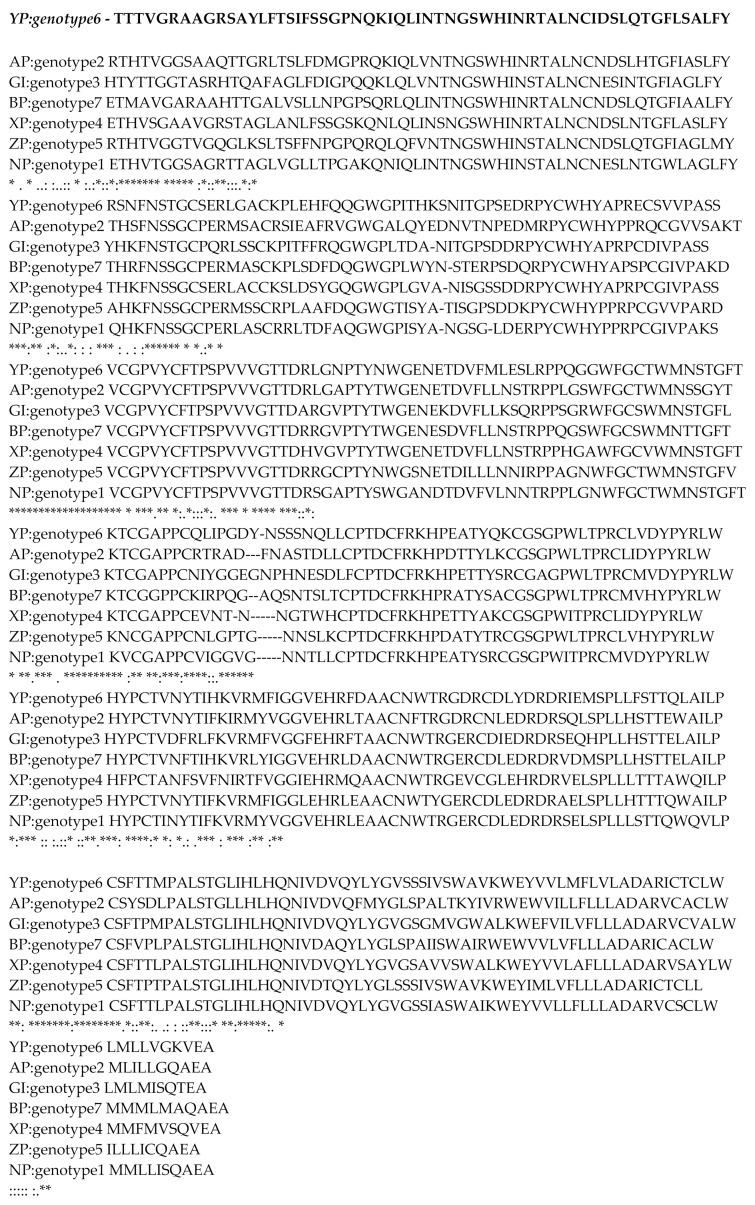
Aligned sequence after multiple alignments.

The template structure used for multiple alignment is 4MWF. ClustalW was used for the purpose of alignment. The alignment obtained is shown in Figure 1. The obtained alignments showed that close similarity exists between template 4MWF and query model. The residues are in comparable positions as shown above. Therefore, they can be expected to have the same function as the template structure. Hence it can be further used for homology modelling.

### 3.2. Model Selection

Different models were obtained after ProCheck analysis. The selected models were listed based on their quality and stereochemical property as shown in Table 1. The tool gave us an insight of the structure, while it can also highlight the portion of the protein that needs to be highlighted. After ProCheck analysis, 23 models were selected on the basis of Ramachandran plot. Computational models were developed in previous studies [2,40] prior to the experimentally determined structures of the E2 glycoprotein. The structures of flavivirus and alphavirus class II fusion proteins were used as modeling templates by investigators. A crystal structure of the E2 glycoprotein of tick-borne encephalitis virus (PDB code 1SVB) [41] presented as the key template for the first of these modeling studies.

The model, having a core value 91.2%, disallowed region 0.0%, maximum deviation value 8.2, bad contacts 6, and generously allowed region 1.0%, was selected for further analysis. 

#### Further Analysis by ProSa

**Figure 2:**
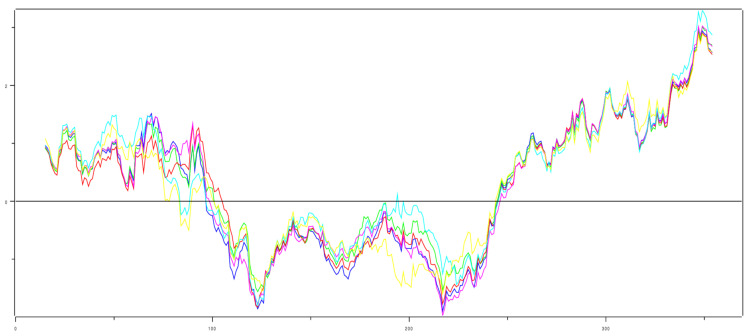
Results of ProSa for selected models.

The selected models were then subjected to further evaluation by ProSa [24]. The tool helped in determining the most suitable model on the basis of energy; therefore, the models having the lowest preference line, as shown in Figure 2, were selected. In the previous investigation [42], scientists predicted that the sequence of the E2 model of HCV would be compared to and conserved as an epitope for vaccine development using in silico approach. During the study, ProSA selected the best model for evaluation. Therefore, the current study shows the importance of these computational tools for studying the best structure. The models obtained through LOMETS [43] were rejected, as they gave no results when they were analyzed with ProSa.

Different colors were given to the models. The overall comparison of the models suggests that MOD29 showed the lowest energy level and is, hence, the most stable confirmation among all other selected models. Therefore, it can be further used as a homology model. The graphical representation suggests that MOD29 has the lowest preference line. 

**Figure 3:**
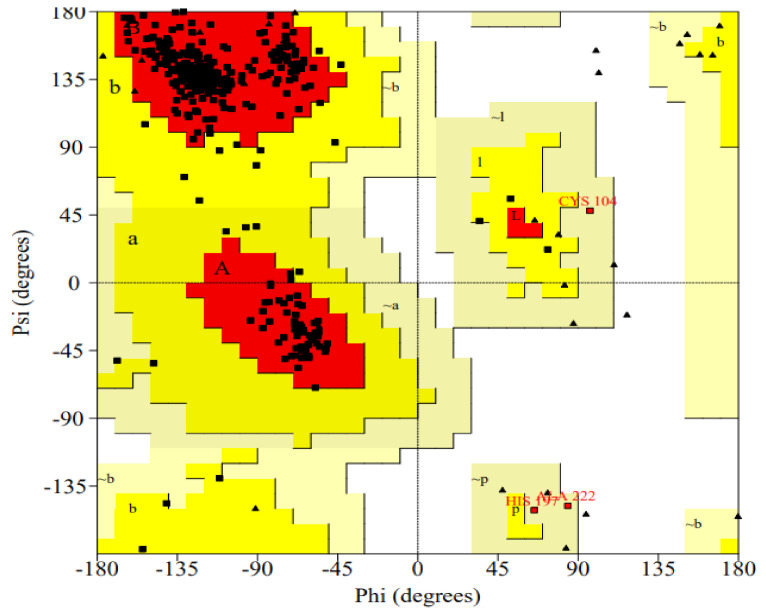
Ramachandran plot of the selected model.

The ProCheck generated Ramachandran plot of the model is shown in Figure 3. The core region contains 91.2% residues, while no residues reside in the disallowed region.

The Ramachandran plot of the model shows that almost 91.2% of residues were present in the most favored region, while no residue was observed in the disallowed region. The number of proline residues was 28. Proline has a specific role in protein splicing, while 32 glycine residues were observed in the model. The 3D model of HCV core protein was designed in a study. The Ramachandran plot of the study reveals that only 87.1% amino acids are present in the favorable region while 12.6% and 0% in allowed and disallowed regions [44]. It can also be predicted from the plot that the high density of amino acids is present in the form of anti-parallel beta sheets, while some of them are in the form of collagen triple helix. Whereas some density of the protein can also be observed in the negative Psi region; therefore, it can be concluded that some of the amino acids can be in the form of right-handed alpha helix.

### 3.3. Model Topology

**Figure 4:**
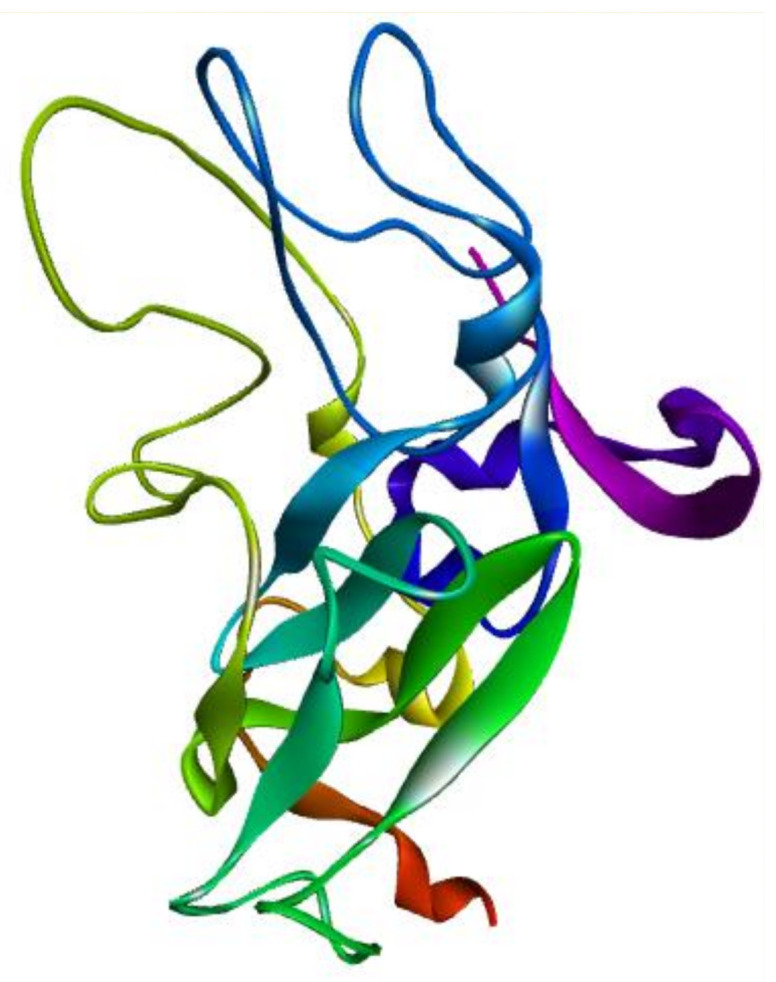
Model of the E2 protein of HCV.

A model of the E2 protein of HCV is shown in Figure 4. The sequence contains 240 amino acids. The 3D model of protein reveals that it contains 11 beta pleated sheets, while 9 alpha helixes were examined during the analysis. The analysis of disulfide bridges was done on a tool (clavius.bc.edu/~clotelab/DiANNA/), which shows that the model has eight disulfide bonds. The protocol used for building disulfide bonds in the model is shown in model patch_ss_template (4MWF). In a study [45], the topology adopted by the specific transmembrane region of HCV envelope proteins has given rise to major controversy, as the model showed less than 30 amino acids. Therefore, current investigations show advancement in model designing of HCV envelope proteins.

### 3.4. Binding Site Analysis

The analysis of the binding site was done by COACH [27]. The web server helps in predicting the binding site with the help of five different tools. Results were based on C-Score value. The residues having the highest C-scores were selected for further analysis. The binding site of HCV envelope protein is also mentioned in the study [46], which can be considered a target site for drug designing. Therefore, the residues observed in the E2 protein by COACH were compared with the whole HCV genome as shown in literature, and only those that were found similar in COACH and the literature were selected. Residues are shown in Table 2. Thus, current findings show the most suitable binding pocket for drug designing.

### 3.5. Virtual Screening on the Basis of Ligands

Three databases were screened using EGCG as a priority model. Obtained ligands were similar to EGCG. Table 3 shows the total ligands during ligand-based virtual screening. Several studies [47,48,49,50] reported virtual screening methods for identification of potential inhibitors for targeting envelope proteins of the flaviviridae family. Thus, current study shows advancement in the virtual screening approach for drug designing against HCV envelope proteins.

### 3.6. Virtual Screening on the Basis of Macromolecule

The protein obtained via homology modeling was used as a model in structure-based virtual screening. Table 4 shows the total ligands during structure-based virtual screening. In a study [51], HCV envelope protein was targeted to inhibit HCV infection and cell to cell transmission by identifying novel drugs through virtual screening. 

### 3.7. PyRx Based Virtual Screening

Ligands obtained from structure-based or ligands-based virtual screening were further assessed via docking by PyRx [37]. The docked ligand-macromolecule complexes were ranked on basis of binding affinity with lowest energy to be at the top. In one of the current studies, computational docking reveals the set of 23 drugs that block the viral infection on CD-81 binding site, but after experimental analysis only one ligand was capable of binding to inhibit the infection of Huh-7 cells. While the binding energy of the drugs ranges from −8.64 to −6.36 [52], the binding energy of the drugs obtained from our virtual screening ranges from −13.2 to −11.

A grid was generated near the binding pockets as predicted by the COACH server. The X, Y, and Z coordinates are characterized in Table 5.

The results obtained through PyRx were further evaluated through LigPlot+ to obtain their interaction with ligand molecules. The 2D models were predicted through LigPlot+. 

### 3.8. Analysis of 2D and 3D Interactions of Docked Complexes

The 3D interactions of the top five molecular docked complexes were analyzed by Discovery Studio Visualizer and the 2D plots for complexes were obtained from LigPlot+, shown in Figure 5, Figure 6, Figure 7, Figure 8, Figure 9, Figure 10, Figure 11, Figure 12, Figure 13 and Figure 14, respectively.

The 2D interaction of mms02387687 ligand and macromolecule suggests that the interaction lacks hydrogen bonding and non-ligand bond interaction, while it represents hydrophobic interactions with some non-ligand residues. The docking scores are 6312. The PyRx results suggest that it has the highest affinity to bind with the macromolecule, i.e., −13.2. Similarly, the 2D interpretation shows that the mms02384293 ligand and non-ligand forms bond with macromolecule residue Pro102 and Ala222, but lack hydrogen bonding. The docking score for the macromolecule is 6332. It has the second-highest binding affinity with the macromolecule after mms02387687, thus having −12.4 binding affinity. The 2D interaction of mms02962350 ligand suggests that it forms the non-ligand bonding interaction with residue Phe64. The docking score is 6676. The results of PyRx suggest that it has −12 binding affinity with the macromolecule. Ligand zinc000150338804 shows two types of bonding such as hydrogen bonding and non-ligand bonding interaction. The hydrogen bonding is present on two sites: Tyr241, which is 3.30cm apart, and Thr52, which is 3.04cm apart from the macromolecule. The non-ligand bonding interaction is also present at two sites, which are Gly177 and Ile94. It is also known as theaflavindigallate. The docking scores are 7982. The PyRx results show that the binding affinity is −12. Ligand zinc000230090738 and macromolecule display non-ligand bonding interaction at two different residues, which are Leu89 and Asp100. Both Leu89 and Asp100 form hydrogen bonding at only one site. The docking score of the ligand is 7342, while the affinity of its binding with the macromolecule is −11.8. Some previous studies [51] show hydrogen and other interactions of sofosbuvir and ribavirin with HCV envelope protein during docking studies. Similarly, in other studies [52], the envelope protein is targeted for identification of various inhibitory molecules. In total, ZINC11882026, ZINC19741044, ZINC00653293, and ZINC15000762 are identified as potential candidates and recognized as appreciable drugs for viral envelope protein. Therefore, current findings suggest there is progress in docking methods for further identification of drugs against HCV envelope protein.

## 4. Conclusions

To reduce the cost of drugs and to limit the amount of time it takes to discover drugs, virtual screening methods are widely used. In this project, E2 protein is modeled through homology modeling. The cell culture analysis of EGCG reveals its affectivity by blocking its path during the inoculation stage. As such, EGCG is used as a standard for screening of potential inhibitors. Structure-based screening approach is successful in obtaining the inhibitors that may behave as a potent target against HCV. Structure-based virtual screening revealed 3700 drugs, while ligand-based virtual screening revealed only 214 drugs. The ligands obtained were finally screened through PyRx and selected on the basis of their binding affinities. Among these ligands, mms02387687 (2-[[5-[(4-ethylphenoxy) methyl]-4-prop-2-enyl-1,2,4-triazol-3-yl]sulfanyl]-N-[3(trifluoromethyl)phenyl] acetamide) was ranked top because of its high binding affinity. The top 5 ligands were further docked with E2 protein. The interaction between the ligands and the protein was analyzed on LigPlot+. We provided valuable information for possible drug-like compounds against HCV and concluded that in-silico dataset might help guide the scientific community toward having a better understanding of ligand molecule interaction with HCV E2 protein.

## Figures and Tables

**Figure 5:**
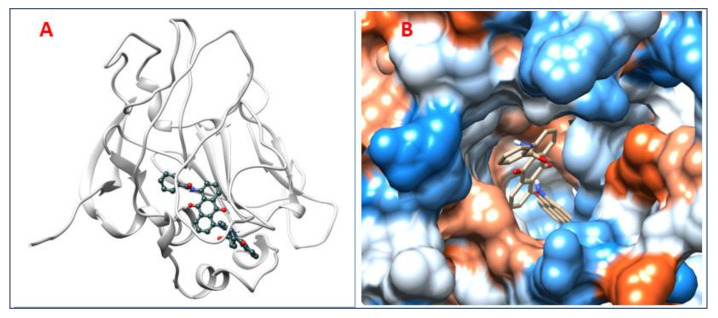
(**A**) 3D interaction of 2-[[5-[(4-ethylphenoxy)methyl]-4-prop-2-enyl-1,2,4-triazol-3-yl]sulfanyl]-N-[3-(trifluoromethyl)phenyl]acetamide and HCV E2 protein having binding affinity −13.2. (**B**) Surface representation of interaction.

**Figure 6:**
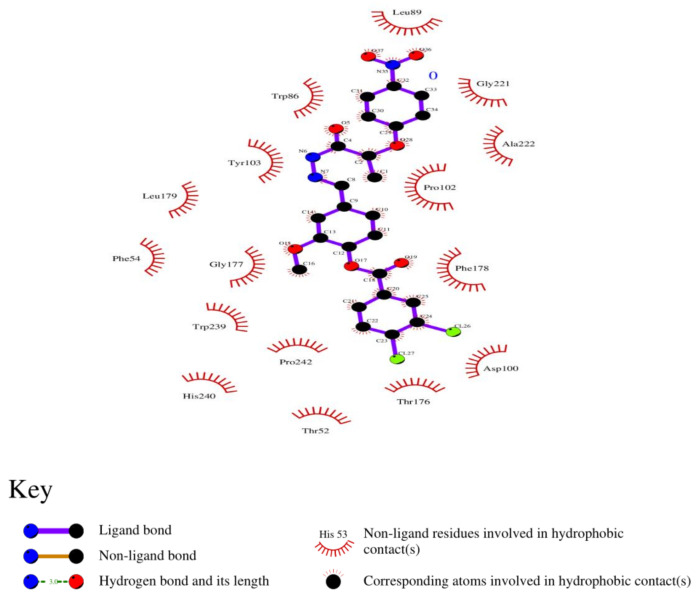
2D interaction of 2-[[5-[(4-ethylphenoxy)methyl]-4-prop-2-enyl-1,2,4-triazol-3-yl]sulfanyl]-N-[3-(trifluoromethyl)phenyl]acetamide and HCV E2 protein.

**Figure 7:**
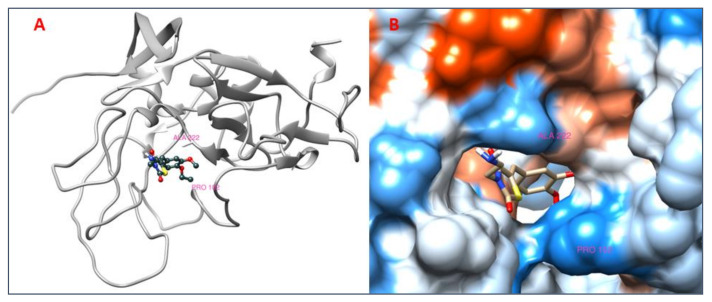
(**A**) 3D interaction of [(5Z)-5-[(4-ethoxy-3-methoxyphenyl)methylidene]-2,4-dioxo-1,3-thiazolidin-3-yl]ethylazanium and HCV E2 protein having binding affinity −12.4; (**B**) Surface representation of interaction.

**Figure 8:**
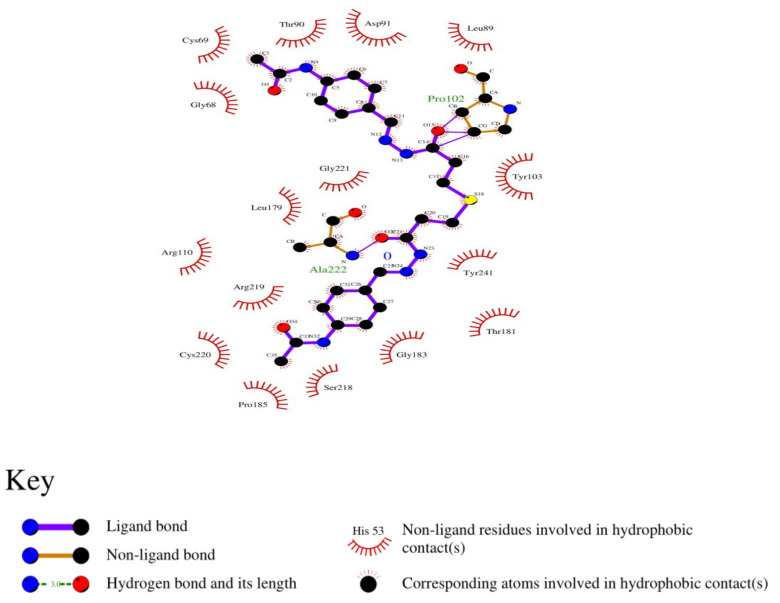
2D interaction of [(5Z)-5-[(4-ethoxy-3-methoxyphenyl)methylidene]-2,4-dioxo-1,3. thiazolidin-3-yl]ethylazanium and HCV E2 protein.

**Figure 9:**
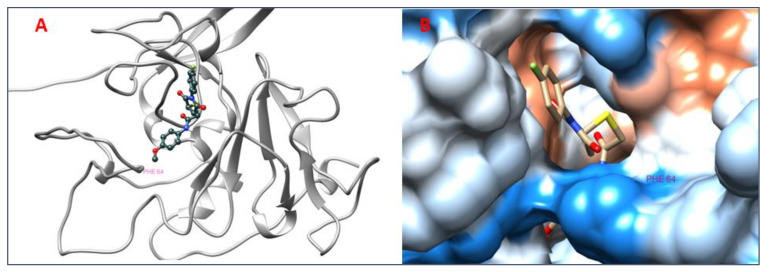
(**A**) 3D interaction of ethyl N-[2,4,6-trioxo-1-(2-phenylethyl)-5-(trifluoromethyl)-7H-pyrrolo[2,3-d]pyrimidin-5-yl]carbamate and HCV E2 protein having binding affinity −12. (**B**) Surface representation of interaction.

**Figure 10:**
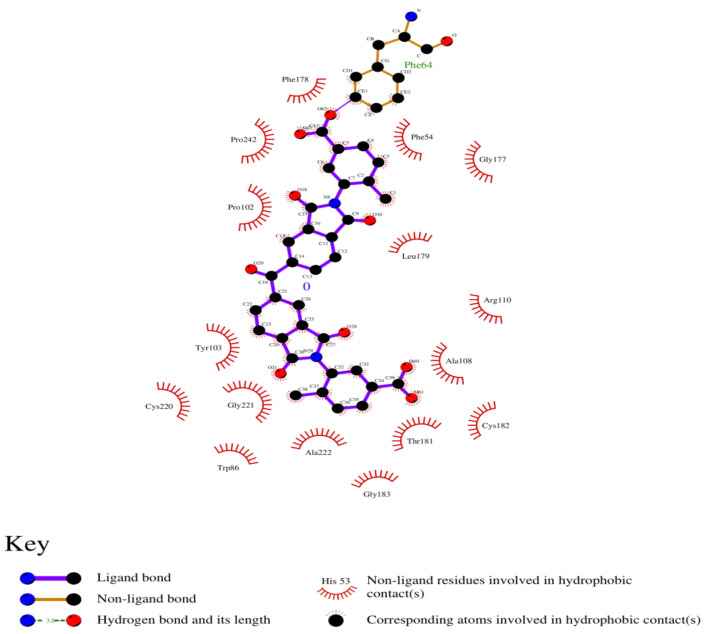
2D interaction of ethyl N-[2,4,6-trioxo-1-(2-phenylethyl)-5-(trifluoromethyl)-7H-pyrrolo[2,3-d]pyrimidin-5-yl]carbamate and HCV E2 protein.

**Figure 11:**
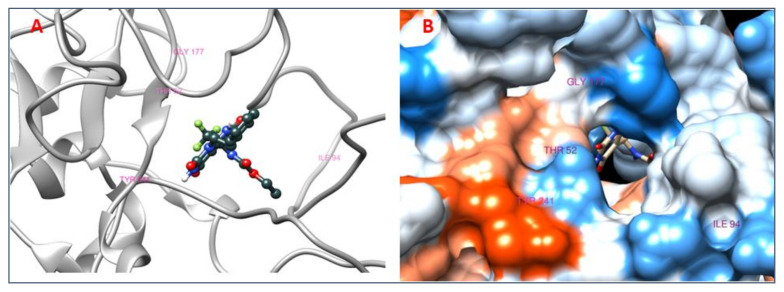
(**A**) 3D interactions of [(2R,3R)-2-[2-[6-[(2R,3R)-5,7-dihydroxy-3-(3,4,5-trihydroxybenzoyl)oxy-3,4-dihydro-2H-chromen-2-yl]-2,3,4-trihydroxyphenyl]-3,4,5-trihydroxyphenyl]-5,7-dihydroxy-3,4-dihydro-2H-chromen-3-yl] 3,4,5-trihydroxybenzoate and HCV E2 protein having binding affinity −12. (**B**) Surface representation of interaction.

**Figure 12:**
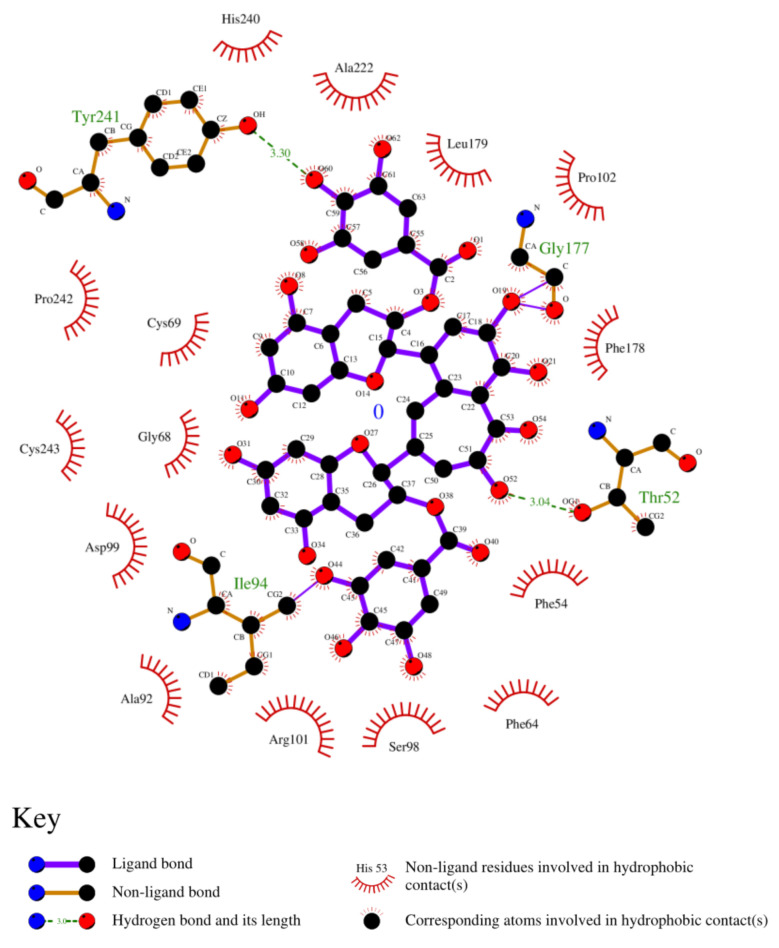
2D interaction of [(2R,3R)-2-[2-[6-[(2R,3R)-5,7-dihydroxy-3-(3,4,5-trihydroxybenzoyl)oxy-3,4-dihydro-2H-chromen-2-yl]-2,3,4-trihydroxyphenyl]-3,4,5-trihydroxyphenyl]-5,7-dihydroxy-3,4-dihydro-2H-chromen-3-yl] 3,4,5-trihydroxybenzoate and HCV E2 protein.

**Figure 13:**
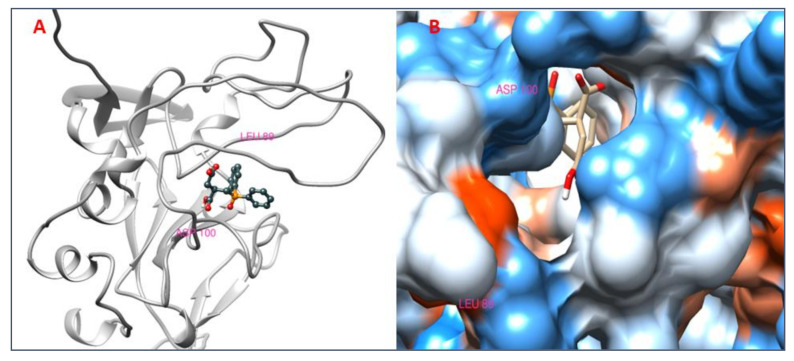
(**A**) 3D interaction of [(2R,3R)-8-[(2R,3R,4R)-5,7-dihydroxy-3-(3,4,5- trihydroxybenzoyl)oxy-2-(3,4,5-trihydroxyphenyl)-3,4-dihydro-2H-chromen-4-yl]-5,7-dihydroxy-2-(3,4,5-trihydroxyphenyl)-3,4-dihydro-2H-chromen-3-yl] 3,4,5-trihydroxybenzoate and HCV E2 protein having binding affinity −11.8. (**B**) Surface representation of interaction.

**Figure 14:**
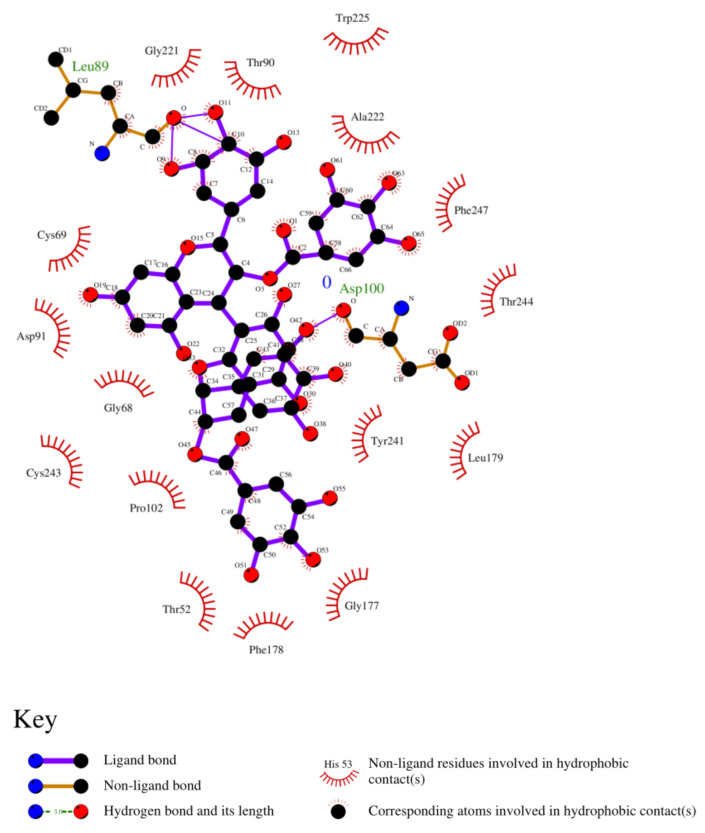
2D interaction of [(2R,3R)-8-[(2R,3R,4R)-5,7-dihydroxy-3-(3,4,5-trihydroxybenzoyl)oxy-2-(3,4,5-trihydroxyphenyl)-3,4-dihydro-2H-chromen-4-yl]-5,7-dihydroxy-2-(3,4,5-trihydroxyphenyl)-3,4-dihydro-2H-chromen-3-yl]3,4,5-trihydroxybenzoate and HCV E2 protein.

**Table 1:** Features of models after ProCheck analysis.

S. no.s	Core	Disallowed Region	Maximum Deviation	Bad Contacts	Generously Allowed Region
**1.**	83.2%	0.0%	4.2	2	1.1%
**2.**	87.8%	0.0%	8.5	2	2.2%
**3.**	87.8%	0.0%	8.5	2	2.2%
**4.**	82.1%	0.0%	3.8	0	2.6%
**5.**	76.7%	0.0%	5.0	0	6.7%
**6.**	83.2%	0.0%	4.2	2	1.1%
**7.**	77.4%	0.0%	4.8	0	6.5%
**8.**	76.7%	0.0%	4.8	0	6.7%
**9.**	82.1%	0.0%	4.8	0	3.6%
**10.**	78.0%	0.0%	4.0	0	2.0%
**11.**	76.7%	0.0%	4.8	0	6.7%
**12.**	91.2%	0.0%	8.2	6	1.0%
**13.**	89.6%	0.3%	4.5	8	1.0%
**14.**	87.3%	0.3%	8.4	11	0.3%
**15.**	88.9%	0.7%	4.7	11	0.3%
**16.**	69.1%	1.0%	5.3	1	5.2%
**17.**	89.6%	1.3%	4.0	6	1.3%
**18.**	77.9%	1.5%	4.7	1	3.8%
**19.**	87.3%	1.6	10.3	6	2.3%
**20.**	66.5%	2.7%	5.3	1	6.4%
**21.**	77.8%	0.0%	7.3	0	3.7%
**22.**	80.0%	0.0%	7.4	0	3.3%
**23.**	75.7%	0.0%	8.4	4	2.2%

**Table 2:** Residues observed in the E2 model of HCV via COACH server and literature.

Number	Residues
1.	412, 413, 414, 415, 416, 417, 418, 419, 420, 421, 422, 423, 524, 525, 526, 527, 528, 529, 530, 535,

**Table 3:** Ligands obtained from different databases during ligand-based virtual screening.

Database	No. of Ligands Obtained
ZINC	100 ligands
PubChem	70 ligands
DrugBank	44 ligands

**Table 4:** Number of ligands obtained in structure-based virtual screening.

Databases	No. of Ligands Obtained
DockBlaster	500
MTI open screen	3000
Pep mms mimic	200

**Table 5:** Grid data for binding site during docking study.

Target Protein	Center Grid Box			Spacing Angstrom	No. of Points in Dimensions			Total Grid Points
	X	Y	Z		X	Y	Z	
E2 Protein	40.038	5.348	53.075	1.00	30	30	32	64,000

## Data Availability

Not applicable.
